# Lightning accidents in the Austrian alps – a 10-year retrospective nationwide analysis

**DOI:** 10.1186/s13049-018-0543-9

**Published:** 2018-09-10

**Authors:** Mathias Ströhle, Bernd Wallner, Michael Lanthaler, Simon Rauch, Hermann Brugger, Peter Paal

**Affiliations:** 1 Department of General and Surgical Critical Care Medicine, Medical University of Innsbruck, Anichstrasse 35, 6020 Innsbruck, Austria; 2 Department of Anaesthesiology and Intensive Care Medicine, Medical University of Innsbruck, Anichstrasse 35, 6020 Innsbruck, Austria; 3 Institute of Mountain Emergency Medicine, EURAC Research, Bolzano, Italy; 4 Department of Anaesthesiology, University Hospital, LMU Munich, Munich, Germany; 5 Department of Anaesthesiology and Intensive Care Medicine, Hospital of the Brothers of St. John of God Salzburg, Teaching Hospital of the Paracelsus Medical University Salzburg, Salzburg, Austria

**Keywords:** Emergency medicine, Electric injuries, Lightning, Lightning injury

## Abstract

**Background:**

Lightning strikes are rare but potentially lethal. The risk for suffering a lightning strike in a mountain environment is unknown. The aim of this nationwide study was to analyse all lightning accidents in the Austrian Alps from 2005 to 2015, to assess the circumstances of the accident, the injury pattern as well as the outcome.

**Methods:**

From 2005 to 2015, data from the national Austrian Alpine Police database as well as the Clinical Information System of Innsbruck Medical University Hospital were searched for the keywords lightning injury, lightning strike, lightning as well as ICD-10 Code T75.0. Additionally, the archive data of Innsbruck Medical University Hospital was searched manually.

**Results:**

The Austrian Alpine Police database, containing 109.168 patients for the years 2005–2015, was screened for lightning accidents. Sixty-four patients had been hit by lightning in the Austrian Alps, 54 were male. Four persons died on scene; survival rate was 93.8%. Two deceased persons were hunters, who were killed by the same lightning strike. Sixty-three patients suffered a lightning strike while doing a recreational activity, mostly hiking (*n* = 55), a few hunting and only one doing occupational timberwork. Sixty-three patients suffered a lightning strike between June and August with nearly half (46.9%) of the accidents happening on a Saturday or Sunday, and mainly (95.3%) between 12:00 and 22:00 h.

**Discussion:**

Persons who perform recreational outdoor and occupational activities in an alpine environment during summer and after noon incur a higher risk of sustaining a lightning strike. The primary risk group includes young male mountaineers and hunters. The mortality rate was low.

**Trial registration:**

The study was approved by the Ethics Committee of the Medical University of Innsbruck (AN4757 315/4.4) and retrospectively registered with Clinical Trials NCT03405467, January 19, 2018.

## Background

Even though the general risk of being struck by lightning is low, it is increased in an outdoor mountain or wilderness environment [1–3]. Worldwide, more than 100 lightning strikes occur each second [3, 4]. Nevertheless, lightning accidents make up only 2–4% of all high-voltage accidents [5]. Lightning may either strike a person outdoors directly or it may be transmitted from a nearby object, such as a tree, a building, through a telephone wire, or even from another person as a contact injury. Even transmission through ground current is possible, since the electric current may enter one leg and exit through the other, due to the potential difference between the two legs [6]. It is regularly reported that more than one patient is injured or killed by the same lightning strike [7]. Contrary to other electrical accidents, victims of lightning have a higher mortality rate, namely 10–30%, and are more severely injured with a 76% risk of long-term sequelae in survivors [4]. The incidence of lightning accidents reported in the literature varies substantially. Hinkelbein et al. reported that in Germany approximately 1000 people are injured by lightning per year [1–3]. In 2003 Holle and López assessed the worldwide impact of lightning, and concluded that 24,000 deaths and 240,000 injuries occur per year. Global mortality due to lightning is estimated at 0.2–1.7 deaths per 1 million per year [6, 8]. However, in most developed countries, the mortality rate of lightning has decreased over the last century and is today on the order of < 0.3 deaths per million people per year. This low fatality rate is due to a decreased amount of labour-intensive agriculture with less work being performed in the open environment and more indoor spare-time habits; lightning strikes during recreational sports and outdoor activities prevail. Rural and agriculture-dominated areas of the world have an annual lightning fatality rate of approximately six deaths per million per year, and this rate is considered applicable to a large portion of the world’s population [9]. In contrast, in most developed temperate and remote areas, lightning is a hazard during outdoor activities, and affects mainly hikers and mountaineers, because weather changes may occur quickly while shelters are not available in the wilderness [2]. The incidence of lightning strikes is notably five times higher in men than in women. To date, a systematic evaluation of medical data on lightning strikes in the mountain environment is missing. The aim of this nationwide study was to analyse all lightning accidents in the Austrian Alps from 2005 to 2015, to assess the circumstances of the accident, the injury pattern as well as the outcome.

## Methods

### Patients

The study was approved by the Ethics Committee of the Medical University of Innsbruck (AN4757 315/4.4) and registered with Clinical Trials NCT03405467. Since November 1, 2005 the Austrian Alpine Police has recorded all emergency calls concerning mountain accidents that were received by the dispatching centre in a database. This database only includes accidents that occur in mountains and remote areas. In the Austrian mountains, all patients affected by a medical emergency will be rescued by a professional team and the Austrian Alpine Police will document the case. Mountain and remote areas are defined as areas where access by standard ambulance vehicles is not possible but achieved by either terrestrial mountain rescue vehicles (usually including four wheel drive) or helicopter rescue (used in > 90% of all mountain and remote rescues). To our knowledge, this results in Europe’s largest database of mountain accidents. The present study covers data from November 1, 2005 to June 13, 2015. The anonymized data from the Austrian Alpine Police were obtained at the site of the accident or after the rescue mission by questioning the rescue team. The complete database of the Austrian Alpine Police was interrogated for lightning accidents and all appropriate cases where extricated by ML. To double-check for patients struck by a lightning in mountains and missed by the Austrian Alpine Police database, we additionally, scanned the Clinical Information System of the Innsbruck Medical University Hospital for i) keywords (lightning and lightning strike), ii) as well as the ICD-10-code T75.0 (effects of lightning and Shock from lightning/Struck by lightning), and iii) manually scanned for patients who had sustained lightning accidents and were admitted to the emergency or trauma department.

### Methods

The collected data included information on the patients’ sex, age and activity; state, altitude, date, day of the week and time of the accident; degree of injury. The degree of injury was subdivided into uninjured, mildly or severely injured and dead. Mild injuries included closed fractures, open wounds without hemodynamically relevant bleeding, arrhythmia or paraesthesia. Severe injuries included traumatic brain injury, open fractures and impairment of the cardiovascular system up to the extent of cardiac arrest. Burns resulting from lightning strike were classified as mild or severe. A lightning accident was classified as an occupational accident when the patient was injured during his job activities. Contrarily, a lightning accident was classified as a recreational accident when the activity had no connection to the patient’s occupation. The data were recorded, processed and statistically analysed with Microsoft Excel 2016 (Microsoft Corporation, Redmond, WA, USA). All personal data were anonymized before processing.

## Results

During the study period the Austrian Alpine Police recorded 38 lightning accidents nationwide with a total of 86 persons. Twelve accidents involved 23 persons who were excluded from the present study, because they did not meet the inclusion criteria: in four cases lightning caused fire without people being involved, three times an emergency call was made in a thunderstorm, four times the word “lightning” was mentioned in the accident report without persons being involved, and once a member of the mountain rescue service was injured while rescuing a lightning victim. One additional patient was identified in the electronic search of the Clinical Information System of Innsbruck Medical University Hospital. Thus, 64 patients hit by lightning were analysed.

Thirty-nine (60.9%) patients involved in a lightning strike were injured in the Austrian states of Tyrol, Salzburg and Vorarlberg. More than two thirds of all Austrian mountain accidents happen in these three provinces. The Innsbruck Medical University Hospital is the level-three hospital for this area. However, no patient in the Austrian Alpine Police database could be matched with the Clinical Information System of Innsbruck Medical University Hospital.

**Fig. 1:**
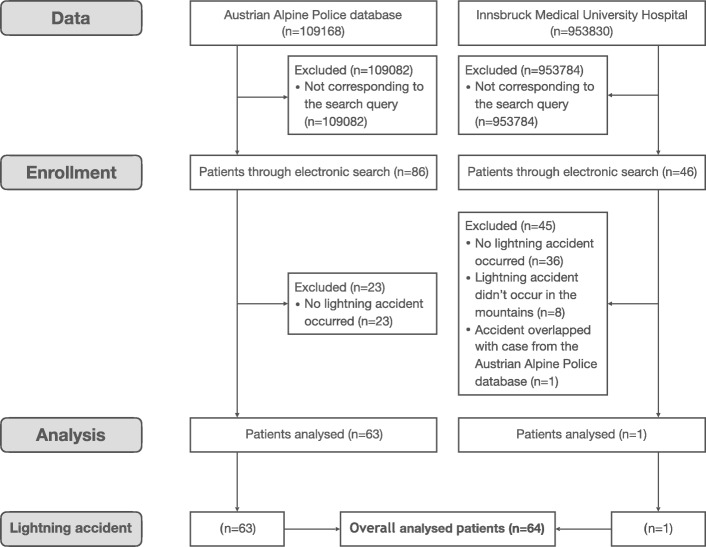
Flow chart showing patient selection, data acquisition, enrolment and analysis for this study following the Consolidated Standards of Reporting Trials (CONSORT) guidelines

We assume these patients were not severely injured, except for the four persons who were found dead at the scene according to police records. A second path of analysis included an ICD-10- (T 75.0) based enquiry of the Clinical Information System at Innsbruck Medical University Hospital. This inquiry identified 10 additional patients, of whom only two were struck by lightning in the mountains. One of these patients was matched with the Austrian Alpine Police database, and it can be assumed that the second accident was not reported through the dispatch centre and therefore not recorded by the Austrian Alpine Police (Fig. 1).

### Demographics

**Fig. 2:**
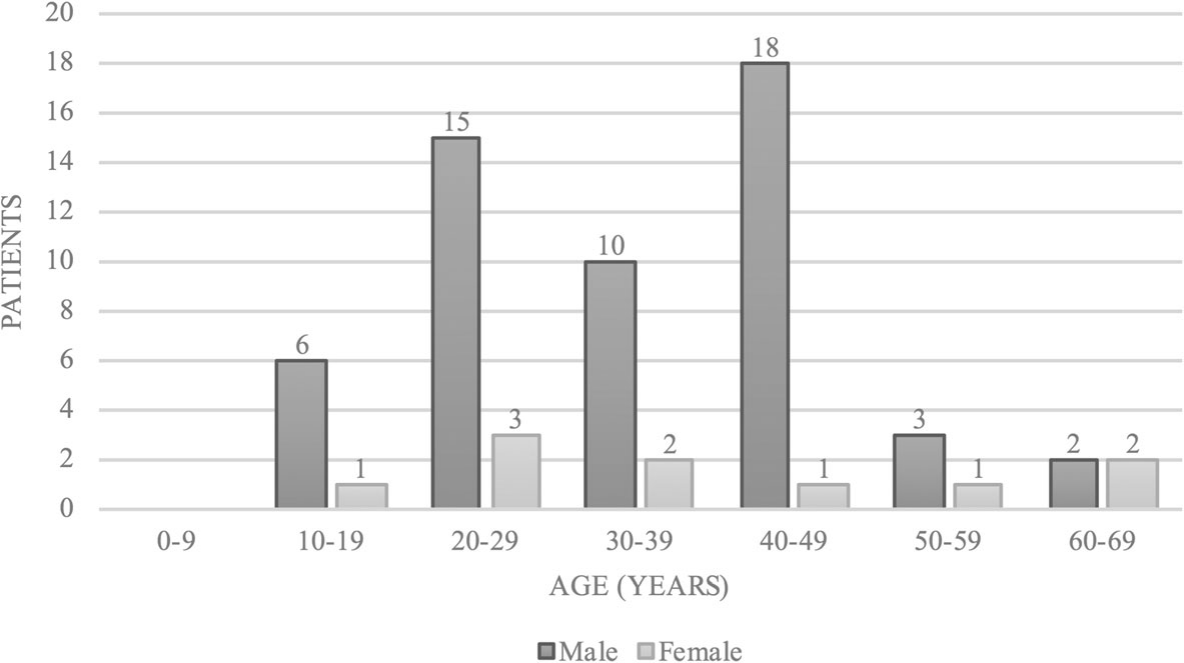
Demographic representation of patient age and sex (*n* = 64)

The 64 patients injured by lightning included 54 males (84.4%) and 10 females (15.6%). Age ranged from 12 to 66 years with a mean of 35 years (Fig. 2).

**Fig. 3:**
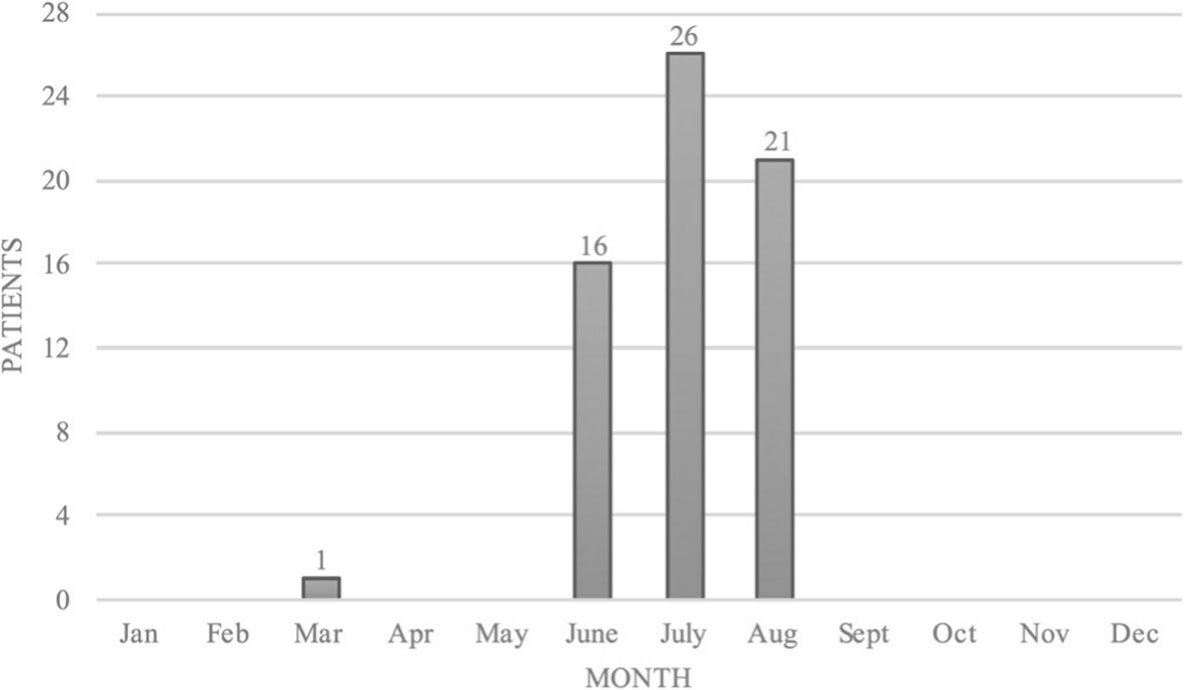
The months in which lightning strikes occurred (*n* = 64)

**Fig. 4:**
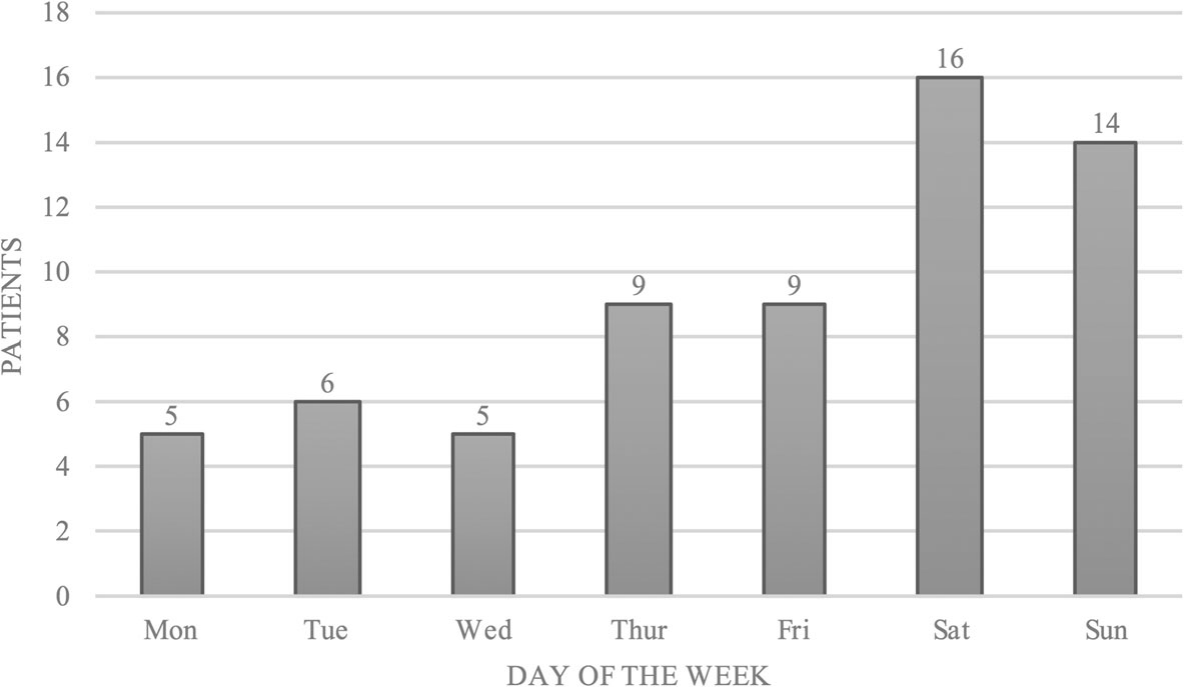
The day of the week on which lightning strikes occurred (*n* = 64)

**Fig. 5:**
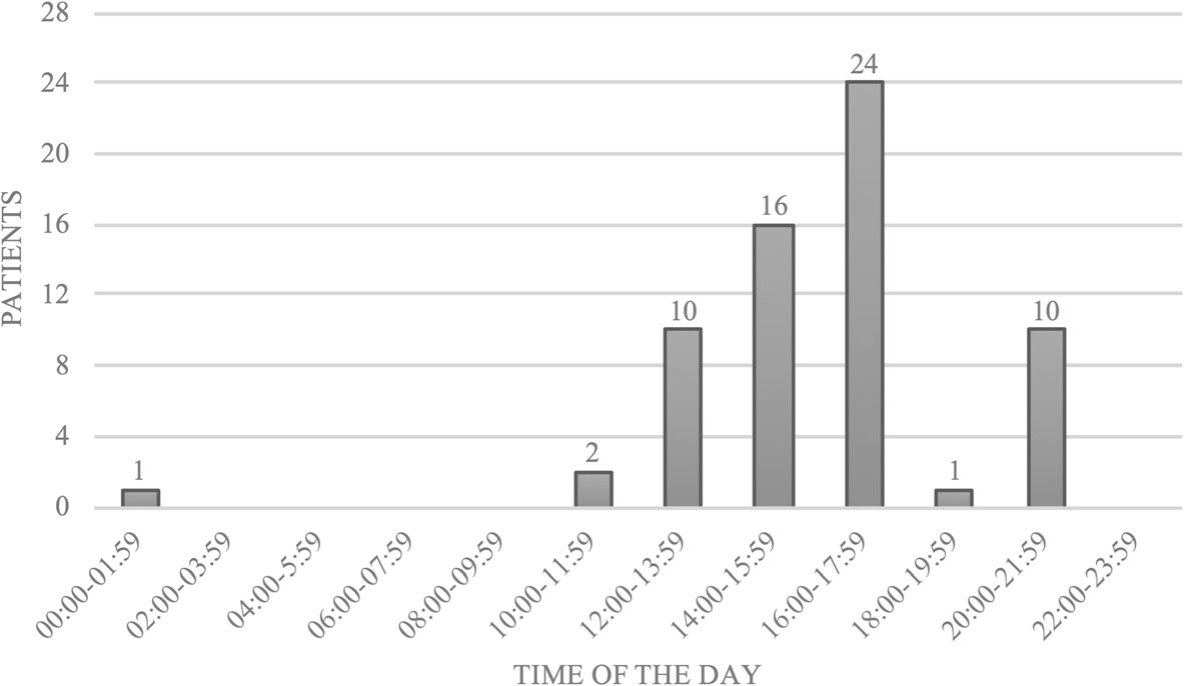
The time of day at which lightning strikes occurred (*n* = 64)

One lightning accident occurred in March (1.6%), all others occurred in June (*n* = 16, 25.0%), July (*n* = 26, 40.6%) and August (*n* = 21, 32.8%), Fig. 3. Lightning accidents occurred on all days of the week with the majority on weekends, i.e. Saturday 16 (25.0%) and Sunday 14 (21.9%), Fig. 4. The vast majority of lightning accidents (95.3%) occurred between 12:00 and 22:00 h, Fig. 5.

**Fig. 6:**
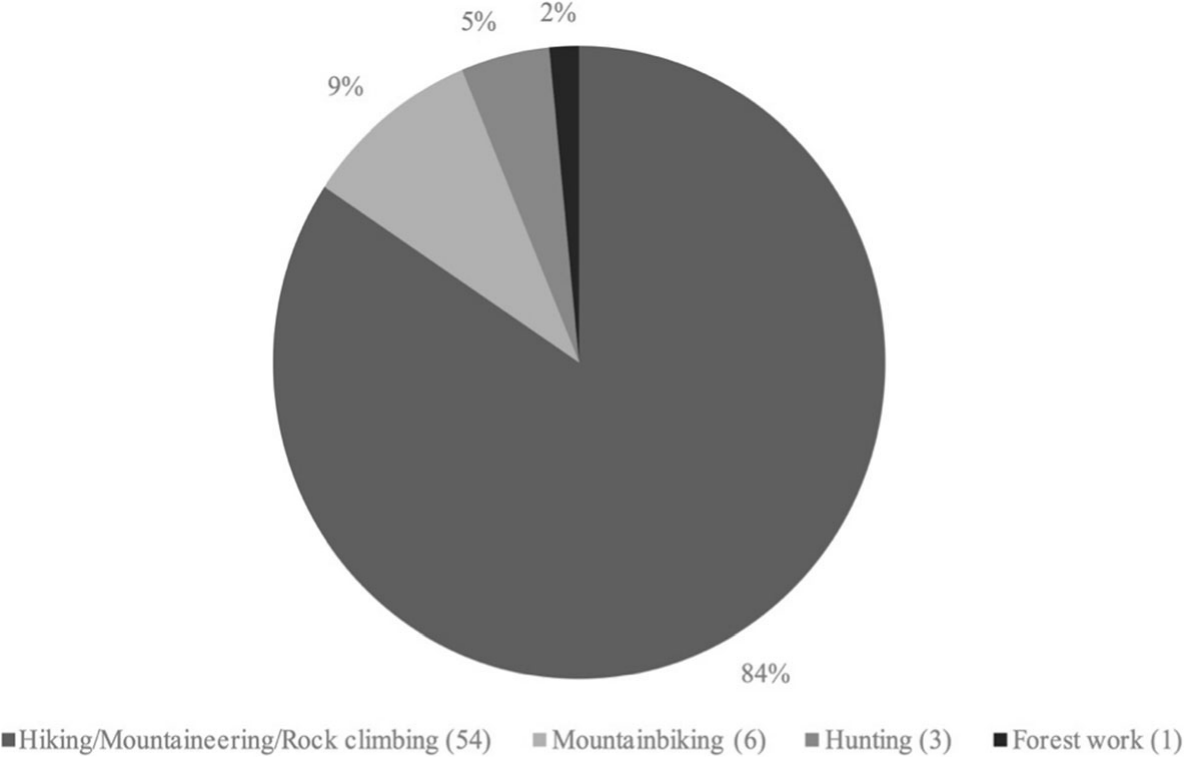
Activity being performed when lightning strikes occurred (*n* = 64)

Of the analysed 64 persons, 63 (98.4%) were hit by lightning during a recreational activity and only one (1.6%) person during an occupational lumberwork activity. Fifty-five (85.9%) persons were hiking, mountaineering or rock-climbing, six (9.4%) mountain biking and three (4.7%) hunting when they were struck by lightning, Fig. 6.

The majority of persons were mildly injured (68.8%), 10.9% were severely injured and 10.9% were uninjured; in two cases (3.1%) no information was provided on the degree of injury. Four (6.3%) persons died; all were male and all died at the site of the accident, which results in a survival rate of 93.8%. Two of the deceased persons were hunters who were taking shelter from lightning in a raised tree or box stand. Both were killed by the same lightning strike. One lumberjack was killed after he finished chopping wood. The fourth person was killed when climbing near a summit. In accordance with other mountain accident statistics from Austria [10], the majority of lightning accidents occurred in the state of Tyrol (*n* = 26, 40.6%), which also has the highest rate of persons performing mountain recreational sports in Austria (31.5%) [11]. The majority of lightning accidents (*n* = 37, 57.8%) occurred at an elevation between 2000 and 2999 m above sea level, 23 accidents (35.9%) at an elevation of 1000–1999 m and two (3.1%) above 3000 m. One person was struck below 1000 m.

## Discussion

The present study reveals that people who undertake occupational and leisure outdoor activities in a mountain environment during the summer months and in the late afternoon have the highest risk of sustaining a lightning accident. The primary risk group includes young male mountaineers and hunters. With four persons dying from a lightning strike in our cohort, the mortality rate of 6.3% is low compared to lightning-associated mortality in the literature [2, 4, 12].

### Incidence and sex aspect of lightning strikes

Men undertake more outdoor activities, and thus their lifetime risk for being struck by lightning is five times higher [4]. In the United States, the calculated lifetime risk for both sexes is 1:12.000 [13] given a life-expectancy of 80 years. For Austria, the general risk of being struck by lightning is expected to be substantially higher due to the higher population density. Furthermore, the risk for experiencing a lightning strike rises with the time spent outdoors and the degree of terrain exposure (e.g. mountain peaks bear a much greater risk than do low-lying and protected areas). The estimated number of unreported cases, for instance uninjured cases, remains unknown, but can only be assumed. In other alpine accidents the number is suspected to be 10-fold higher, as known from avalanche statistics. Similar reports of misleading number are found in other studies [14]. In the current study, considerably more men were injured or killed by lightning than were women (84.4% vs. 15.6%). One possible explanation might be that more men than women spend time in the mountains and may also take greater risks and seek shelter at a later stage [15]. Similarly, according to an analysis conducted by the Austrian Board of Alpine Security, 87% of all accidents in the mountains involve men [16]. However, the overall risk involved in mountain sports is difficult to estimate since there are only limited data on the percentage of men and women indulging in mountain sport activities. In general, the age distribution for lightening accidents is consistent with the population pyramid, but an age analysis of both males and females in our study shows two peaks, one from 20 to 29 years and another from 40 to 49 years. Rock climbing is very popular in the first age group, while the second group tends to enjoy mountaineering and hiking. A study performed by Cherington et al. also analysed the risk of being struck by lightning outdoors while performing sports in various surroundings and reported that mountain climbers are prone to a greater risk of being struck by lightning [17].

### Time and date of lightning strikes

The greatest risk for sustaining a lightning strike is given in summer, more specifically in the afternoon. At that time, the meteorologically strongest lightning activity coincides with the largest number of people in mountain terrain. Being on a mountain in the afternoon entails great danger for mountaineers and climbers, since they may have been physically active for hours, may be tired and still far from safe shelter. Cherington et al. reported that the highest frequency of lightning strikes in the Rocky Mountains occurs between 11:00 and 21:00 from April to September [17]. Most lightning strikes in Austria occur from June to August with a peak in July after noon. Furthermore, most lightning strikes occurred on weekends, which are naturally most popular for alpine outdoor activities enjoyed by the working population.

### Injury severity and survival

People who hike and rock climb have a high risk for lightning strikes. In mountain terrain, even an indirect lightning strike that extends into a shock-wave or that causes abrupt muscle contraction can lead to severe injuries secondary to fall or stumble. Rock climbers were described to have fallen off a wall and have been injured or died because of severe accidents associated with high-energy impact [2]. In the present study, two-thirds of patients survived the lightning strike with mild injuries Table 1.) In a review, Zafren et al. comment on the lightning hazard during outdoor activities in mostly temperate remote areas and also state that lightning accidents often result in only mild injuries [2]. Typical injuries after a lightning strike are burns to the skin. Due to the extremely short duration of the electric impulse and the surface effect, burns are typically of first and second degree.

**Table 1:** Injury suffered when hit by lightning. Mild injuries included closed fractures, open wounds without hemodynamically relevant bleeding, arrhythmia or paraesthesia

	Uninjured	Mildly injured	Severly injured	Dead	Unknown
Acute stress reaction	3	6			
Cardiovascular disorder		13	2	3	
Cardiovascular disorder and TBI			1		
Cardiovascular disorder and TBI, open wound head			1		
Burn injury, whole body		1			
Burn injury, shoulder		3			
Burn injury, upper arm		1			
Burn injury, forearm		1			
Burn injury, foot		2			
Burn injury ankle joint, multiple lacerations lower leg			1		
Injury not further specified, forearm		4			
Injury not further specified, hand		1			
Open wound, head		2			
Open wound, lower leg (due to crampon)		1			
Fracture, shoulder			1		
Fracture, lower leg		1			
Paresthesia lower arm, vertigo		1			
Parethesia not further defined		1			
Unknown injury pattern, not further specified by emergency service on scene	4	6	1	1	2
	7	44	7	4	2

Severe injuries included traumatic brain injury, open fractures and impairment of the cardiovascular system up to the extent of cardiac arrest

The surface effect describes the phenomenon that the main part of the lightning current is transmitted along the body surface. The high electric resistance of the skin causes a significant drop in voltage and explains why lightning strikes can be survived [18]. The degree of injury depends on the electric current of the lightning strike and the duration of the impact. Patient-related factors are the condition of the contacted surface (i.e. thickness and humidity of the skin) and the path the electric current takes through the body.

Zafren et al. state that for hikers and mountaineers, it may be particularly difficult to find a safe place in the wilderness and conclude that lightning injuries are largely avoidable. These findings are comparable to our and other similar studies [2]. Weichenthal et al. conducted a study in the US national parks and demonstrated that there are insufficient levels of awareness, for instance lightning safety awareness was higher in regular national park visitors than individuals from the metropolitan community [19].

In 2001 Burtscher M. et al. stated that about 10 million hikers and skiers are present annually in the mountains of Austria and about 40 million in the whole European Alps. The number of tourist night stops increased approximately 27% from 2001 to 2017. We conclude that approximately 12.7 million people are active in the Austrian mountains per year (the numbers split almost equally between summer and winter). With about 6.35 million people in the mountains in summer time and 64 persons hit by lightning in ten years (6.4/year) we assume a relative risk of about 1:1 million to be hit as an individual per year. The probability to be lethally hit by lightning is less than 1:16 million in summer and less than 1:30 million all year round.

The survival rate in our study (93.8%) was exceptionally high, while two other studies reported an average survival rate of only 70% [2, 12]. The reasons for the low mortality amongst those who were struck by lightning remain hypothetical. First, mountaineers are on average more informed than the urban or rural population. They may be more aware of situations, which incur a high risk of death in case of a lightning strike, e.g. seeking cover under a tree, carrying metal gear. Second, they may limit the impact of a lightning strike on their body by adopting the single-contact position and by wearing outdoor clothing which may keep the electrical current flow on the outside of the body instead of flowing through the vulnerable heart and thereby causing cardiac arrest.

We assume that our low mortality rate as compared to other studies was due to individual persons or groups of only a few people being involved in the lightning events. Maybe this reflects the fact that mountaineers and climbers rarely appear in large groups and usually keep a distance from each other. Therefore, the risk for a mass-casualty incident second to lightning is rarely found in the mountains [20].

Only one fatal lightning strike occurred while doing forest work, although rural or agricultural work is one of the biggest risk factors for fatal lightning accidents. We therefore conclude that the mortality rate of the analysed population can hardly be compared to that of previous studies.

All four deceased persons were already dead by the time the rescue team arrived. Therefore, a direct lightning strike must be assumed. Three of the four lethal injuries occurred in densely forested areas. Trees, like similar shelters (e.g. pavilion, rain shelter and tent) do not provide adequate protection from lightning and may even attract a lightning strike [21, 22]. Similarly, other studies reported that nearly all fatalities occurred while the victims were in open terrain, with the majority occurring under trees. A great number of lightning accidents involved people who sought protection from the storm under “shelters” that provide no protection from lightning [22, 23].

Finally, people who indulge in mountaineering tend to lead a healthier lifestyle, which also may cause less cardiac vulnerability and less susceptibility towards ventricular fibrillation or other arrhythmias. Lightning strikes are an exceptional form of electrocution because of their extremely high amperage of up to 100.000 A [3, 4], high voltage of about 0.3–2.2 million volts [24–26] and extremely short conduction time of 0.02–100 milliseconds [3, 25, 26], which, taken together, result in very high temperatures of 25.000 °C - 30.000 °C. The most common cause of death is cardiac arrest due to ventricular fibrillation and subsequent asystole [27, 28].

Unfortunately, bystander CPR was not performed in any incident. The reasons for this are not evident. The continuous current can paralyze the respiratory centre of the brain, cause prolonged apnoea and lead to secondary hypoxia and cardiac arrest. To resolve this life-threatening situation, continuous and prolonged cardiopulmonary resuscitation must be performed. If multiple persons are struck by lightning, contrary to the rules for triage in the case of a non-lightning accident, priority is given to the person without vital functions [29, 30]. Davis et al. and the Wilderness Medical Society provided practice guidelines for treatment in 2012 and gave an update on this topic in 2014, here they advise to seek shelter once thunder can be heard, avoid ridgelines or summits. They also advise people to take the lightning position, which is a position that involves sitting or crouching with knees and feet close together to create only one point of contact with the ground [31, 32].

Furthermore Davis et al. even provide guidelines to prevent lightning accidents in a Mountain Environment: they recommend avoiding peaks and ridgelines in the afternoon as thunderstorms are most frequent during this time period. If caught in a thunderstorm, climbers should tie-off individually as lightning is able to conduct over wet climbing ropes and may affect both climber and bilayer. Individuals should discard metal objects such as ski poles or mountaineering axes to avoid contact burns [31].

The current study has several limitations. Firstly, the data obtained from the Austrian Alpine Police could not be matched with hospital data to obtain more medical information on the lightning victims. This is mainly due to the multicentre treatment of lightning victims in various hospitals and also to the anonymization of patient data. Secondly, we believe a large number of cases go unreported, especially in the case of an indirect lightning strike and when patients remain uninjured. Unfortunately, this can hardly be proven and the literature currently contains no information on this.

## Conclusions

The present study reveals that people who undertake occupational and leisure activities outdoors in an Alpine environment during the summer months and in the late afternoon expose themselves to a considerably greater risk of sustaining a lightning accident. The primary risk group is young male mountaineers and hunters, who show quite different demographics and a very low mortality rate. Better education and prevention measures can help prevent most lightning accidents in Austria’s mountains. This study shows that lightning accidents with human participation should be better linked to medical databases in hospitals in order to gain a better insight into the accident mechanism and outcome.

## Abbreviations

CPR: Cardiopulmonary resuscitation
GPS: Global Positioning System
ICD: International Statistical Classification of Diseases and Related Health Problems

## References

[1] Leikin JB (1997). Environmental injuries. Dis Mon.

[2] Zafren K (2005). Lightning injuries: prevention and on-site treatment in mountains and remote areas. Official guidelines of the International Commission for Mountain Emergency Medicine and the medical Commission of the International Mountaineering and Climbing Federation (ICAR and UIAA MEDCOM). Resuscitation.

[3] Hinkelbein J, Spelten O, Wetsch WA (2013). Lightning strikes and lightning injuries in prehospital emergency medicine. Relevance, results, and practical implications. Unfallchirurg.

[4] Ritenour AE (2008). Lightning injury: a review. Burns.

[5] Dokov W (2009). Assessment of risk factors for death in electrical injury. Burns.

[6] Forster SA (2013). Lightning burn--review and case report. Burns.

[7] Kubilius D, Rimdeika R (2012). Simultaneous lightning injury in a group of people: case report. Burns.

[8] Wetli CV (1996). Keraunopathology. An analysis of 45 fatalities. Am J Forensic Med Pathol.

[9] L., H.R., Annual rates of lightning fatalities by country, in Internet 2008. p. S. 21–3.

[10] Österreichisches Kuratorium für Alpine Sicherheit, A.Ö., *Alpine Unfallforschung*, in https://www.alpinesicherheit.at/data/docs/2016/2016-05-25%20Alpinunfallstatistik_2014_15.pdf2015.

[11] Österreichisches Kuratorium für Alpine Sicherheit, A.Ö., *Alpine Gesamtunfallstatistik 2014*, 2014.

[12] Cooper MA (1980). Lightning injuries: prognostic signs for death. Ann Emerg Med.

[13] NLSH, P., NWS Lightning Safety Home Page.

[14] Thomson EM, Howard TM (2013). Lightning injuries in sports and recreation. Curr Sports Med Rep.

[15] JS., J.J. A detailed analysis of lightning deaths in the United States from 2006 through 2013. 2015.

[16] Österreichisches Kuratorium für Alpine Sicherheit, A.Ö., *Auswertung Alpinunfalldatenbank*.

[17] Cherington, M., Closing the gap on the actual numbers of lightning casualties and deaths*.* American Meteorological Society, 1999. 11th conference on applied climatology**,** Dallas, January 10-15**,** 1999.

[18] Cwinn AA, Cantrill SV (1985). Lightning injuries. J Emerg Med.

[19] Weichenthal L (2011). Lightning safety awareness of visitors in three California national parks. Wilderness Environ Med.

[20] Blancher M, et al. Management of Multi-Casualty Incidents in mountain rescue: evidence-based guidelines of the International Commission for Mountain Emergency Medicine (ICAR MEDCOM). High Alt Med Biol. 2018;19(2):131–40. 10.1089/ham.2017.0143 PMC6014052 29446647

[21] Holle RL (1995). Safety in the presence of lightning. Semin Neurol.

[22] DJ., C., Preventing Suddent Death in Sport and Physical Activity Second Edition ed 2017: American Journal of Sports Medicine.

[23] RL., H., Rank of could-to-ground flash densities by state from 2004–2013. , in National Lightninig Detection Network 2015.

[24] Delaney JS, Drummond R (2002). Mass casualties and triage at a sporting event. Br J Sports Med.

[25] Maghsoudi H, Adyani Y, Ahmadian N (2007). Electrical and lightning injuries. J Burn Care Res.

[26] Soar J (2015). European resuscitation council guidelines for resuscitation 2015: section 3. Adult advanced life support Resuscitation.

[27] Zack F (1997). Myocardial injury due to lightning. Int J Legal Med.

[28] Scantling D (2016). Inducing therapeutic hypothermia in cardiac arrest caused by lightning strike. Wilderness Environ Med.

[29] Cooper MA (1983). Lightning injuries. Emerg Med Clin North Am.

[30] Spano SJ (2015). A lightning multiple casualty incident in Sequoia and kings canyon national parks. Wilderness Environ Med.

[31] Davis C (2014). Wilderness medical society practice guidelines for the prevention and treatment of lightning injuries: 2014 update. Wilderness Environ Med.

[32] Davis C (2012). Wilderness medical society practice guidelines for the prevention and treatment of lightning injuries. Wilderness Environ Med.

